# Clinical and pathological heterogeneity of four common fusion subtypes in Xp11.2 translocation renal cell carcinoma

**DOI:** 10.3389/fonc.2023.1116648

**Published:** 2023-02-03

**Authors:** Wei Guo, Yiqi Zhu, Xiaohong Pu, Hongqian Guo, Weidong Gan

**Affiliations:** ^1^ Department of Urology, Drum Tower Clinical Medical School of Nanjing Medical University, Nanjing, Jiangsu, China; ^2^ Department of Urology, Taizhou People’s Hospital Affiliated to Nanjing Medical University, Taizhou, Jiangsu, China; ^3^ Department of Urology, Nanjing Drum Tower Hospital, The Affiliated Hospital of Nanjing University Medical School, Nanjing, Jiangsu, China; ^4^ Department of Pathology, Nanjing Drum Tower Hospital, The Affiliated Hospital of Nanjing University Medical School, Nanjing, Jiangsu, China

**Keywords:** Xp11.2 translocation renal cell carcinoma, TFE3, FISH, DBHS family, prognosis

## Abstract

**Background:**

Xp11.2 translocation renal cell carcinoma (Xp11.2 tRCC) is a group of rare and highly heterogeneous renal cell carcinoma (RCC). The translocation involving TFE3 and different fusion partners lead to overexpression of the chimeric protein. The purpose of this study is to explore the clinicopathological features of Xp11.2 tRCC with four common fusion subtypes.

**Methods:**

We screened out 40 Xp11.2 tRCC patients from January 2007 to August 2021 in our institution. The diagnosis was initially confirmed by TFE3 immunohistochemistry (IHC) and fluorescence *in situ* hybridization (FISH) assay and their fusion partners were verified by RNA sequencing. Then the 40 cases were divided into two groups (DBHS family and non-DBHS family group) and a clinical comparison among the four common fusion subtypes was performed.

**Results:**

Among the 40 cases, 11 cases with SFPQ-TFE3 gene fusion and 7 cases with NONO-TFE3 gene fusion were classified in DBHS group, the remaining cases with ASPL-TFE3 (11 cases) or PRCC-TFE3 (11 cases) gene fusion were classified in non-DBHS group. Lymph node (LN) metastasis (P=0.027) and distant metastasis (P=0.009) were more common seen in non-DBHS family group than DBHS family group and cases in DBHS family group have better progressive-free survival (PFS) (P=0.02). In addition, ASPL-TFE3 fusion was associated with worse outcome (P=0.03) while NONO-TFE3 fusion (P=0.04) predicted a better prognosis.

**Conclusions:**

Different fusion partner genes may play a functional role in various morphology, molecular and biological features of Xp11.2 tRCCs. The impact of fusion partners on clinical characteristics of Xp11.2 tRCCs deserves further exploration.

## 1 Background

Xp11.2 translocation renal cell carcinoma (Xp11.2 tRCC) is a rare and distinct subtype of RCC, classified in Microphthalmia (MiT) transcription factor family translocation renal cell carcinomas (RCC) in 2016 (1). The most notable feature of Xp11.2 tRCC is chromosome translocations involving TFE3 gene, resulting in fusion with various gene partners (2). Since the discovery of ASPL-TFE3 as the first gene fusion in Xp11.2 tRCC (3), the number of fusion partners has expanded with the development of next-generation sequencing (NGS) technologies. The relatively common fusion genes included ASPSCR1 (ASPL), PRCC, NONO (p54nrb), SFPQ (PSF) and other rare fusion genes such as CLTC, RBM10, MED15, SETD1B, ZC3H4, LUC7L3, KHSRP, PARP14, DVL2, GRIPAP1 were occasionally reported (3–13). The diversity of the fusion partners drastically affects biological behaviors and chromosome structures, which in turn leads to the clinical heterogeneity of Xp11.2 tRCC (14). However, due to the rarity of Xp11.2 tRCC, more cases with definite fusion types are needed to compare the clinical characteristics of Xp11.2 tRCC.

The impact of fusion genes on Xp11.2 tRCC is multifaceted. The common fusion partners of Xp11.2 tRCC, SFPQ and NONO, are both members of the drosophila behavior/human splicing (DBHS) protein family and participate in almost all steps of gene regulation (15–18). The protein products encoded by DBHS family are functionally conserved and largely considered as nuclear factors (15). Previous study has suggested SFPQ and NONO may play an important role in nuclear localization of TFE3 during tumor progression(2). In addition, the inversion of the TFE3 and NONO results in an equivocal split signal distance in fluorescence *in situ* hybridization (FISH), which makes it difficult to diagnose Xp11.2 with this special fusion type (19). On the other hand, another common fusion partner, ASPL, was proved to be associated with aggressive behavior and poor prognosis compared with other fusion genes (12, 14). PRCC can bind to MAD2B (a mitotic checkpoint protein) directly and regulate mitosis, but this interaction can be impaired by the translocation of PRCC and TFE3 (20). As most molecularly confirmed Xp11.2 tRCC cases was described in small series, so far, there are few reports about systematic clinical comparison of Xp11.2 tRCC with common fusion types.

In the present study, we identified 40 cases of Xp11.2 tRCC with four common fusion types (ASPL, PRCC, NONO and SFPQ) by RNA sequencing and described their typical morphological and molecular features. Furthermore, we divided the 40 cases into two groups (DBHS family and non-DBHS family group) and compared their clinicopathological characteristics and prognosis.

## 2 Methods

### 2.1 Patients and samples

In this study, 40 cases with suspicious morphological features of Xp11.2 tRCC were retrieved from the diagnostic files in Nanjing Drum Tower Hospital between January 2007 to August 2021. The hematoxylin & eosin (H&E) slides were reviewed independently by two specialist uropathologists and the diagnosis of these cases was based on preliminary TFE3 immunohistochemistry (IHC) or FISH assay. Some of these cases have been reported in the previous literature and their fusion partners have been confirmed by reverse transcriptase polymerase chain reaction (RT-PCR) (21, 22). The available clinicopathological features and follow-up data were recorded. The TNM stage and nuclear grade were classified by the AJCC 2017 TNM Staging System and WHO/ISUP grading system, respectively.

### 2.2 TFE3 IHC

The 4-μm-thick sections were prepared from 10% FFPE tissue blocks for TFE3 IHC staining. All slides were exposed to 3% H_2_O_2_ for 10 minutes at room temperature to block endogenous peroxidase activity. TFE3 (HPA023881, Sigma, USA), cathepsin K (ab19027, Abcam, Cambridge, UK), CD10 (ab227640, Abcam, Cambridge, UK), CA-IX (ab107257, Abcam, Cambridge, UK), Vimentin (ab8978, Abcam, Cambridge, UK), CD117(ab32363, Abcam, Cambridge, UK), CK7 (ab181598, Abcam, Cambridge, UK) antibody were incubated with tumor sections in a humidified chamber at 4°C overnight, then the anti-mouse or anti-rabbit peroxidase-conjugated secondary antibody (EnVision™ Detection Kit, DAKO, Denmark) were used with the sections at 37°C for 30 minutes.

The result was evaluated in a semiquantitative manner by multiplying the staining intensity (0 = no staining, 1 = mild staining, 2 =moderate staining, and 3 = strong staining) by the percentage of immunoreactive tumor cells (0–100). The final immunostaining result was calculated as following: negative (0), score <25; weak positive (1+), score 26–100; moderate positive (2+), score 101–200; strong positive (3+), score 201–300.

### 2.3 Fluorescence *in situ* hybridization

FISH assay was performed on 3-μm -thick FFPE tissue sections with TFE3 positive immunostaining. The commercial dual-color break-apart FISH probes (LBP, Guangzhou, China) were used to detect TFE3 gene arrangement. The telomere and centromeric sides were labeled with 5-ROX-dUTP (red) and fluorescein-12-dUTP (green), respectively. Briefly, the FFPE sections were deparaffinized and permeabilized after a series of treatments, then the probes were applied to the tumor region. All the slides containing the tissue DNA probes denatured at 85°C in a *in situ* thermocycler for 5 minutes and hybridized at 37°C overnight. After washing in 2×SSC for 10 minutes and in 0.1% NP-40/2×SSC for 5 minutes at room temperature, the slides were air dried and 5 μL of 4′,6-diamidino-2-phenylindole (DAPI) was used to counterstain the nuclei. The detail FISH protocol has been reported previously. Routinely, at least 100 non-overlapping nuclei were counted under Olympus BX51TRF fluorescence microscope (Olympus, Tokyo, Japan) at ×1000 magnification. The signals separated by a distance >2 signal diameter was considered to be split. For cases with suspicious NONO-TFE3 fusion (equivocal FISH pattern), RNA sequencing was performed to verify the result. When >10% of the nuclei showed evidence of split signals, the result was considered to be positive.

### 2.4 RNA sequencing

The 40 cases with positive FISH results were analyzed by RNA sequencing. Total RNA from FFPE samples was extracted using RNeasy kit (QIAGEN). RNase H was used to depleted ribosomal RNA and KAPA Stranded RNA-seq Kit with RiboErase (HMR) (KAPA Biosystems) was used to library preparation. Library concentration and library quality was accessed by KAPA Library Quantification Kit (KAPA Biosystems) and Agilent High Sensitivity DNA kit on Bioanalyzer 2100 (Agilent Technologies), respectively. Then the products were sequenced on Illumina HiSeq next-generation sequencing (NGS) platforms (Illumina).

### 2.5 Statistical methods

Statistical analyses were conducted by SPSS software version 26.0 (SPSS, Inc., Chicago, IL, USA) and figures were depicted by GraphPad Prism software version 8.0 (GraphPad Software, San Diego, CA). Student’ s t test or Mann–Whitney U test or was performed to compare continuous data. Pearson chi-square test or Fisher’ s exact test was performed to compare categorical data. Kaplan– Meier method was used to compare the survival data, and statistical comparisons between the two groups were evaluated with Log-rank test. A two-sided P < 0.05 was considered statistically significant.

## 3 Results

### 3.1 Clinicopathologic features

The detailed clinicopathologic features of the 40 Xp11.2 tRCC patients are shown in **Table 1**. Xp11.2 tRCC most commonly occurred in young adults, at a median age at diagnosis of 35.5 years and a mean of 37.3 years, ranging from 7 to 70 years. The incidence rate was slightly higher in female than that in male, with a male: female ratio of 1:1.4. The median tumor size was 4.5cm and the mean size was 5.5cm, respectively. Regional lymph node metastasis was found in 9 patients (22.5%) at diagnosis and distant metastasis had developed in 13 patients (32.5%) at the last follow‐up. 31 patients (77.5%) were at earlier pT stage (T1-T2) while 9 patients (22.5%) were at an advanced stage (T3-T4) at the time of diagnosis. Higher nuclear grades (WHO/ISUP grade: 3-4) was observed in more than half the patients (60%). 10 (25.0%) patients died at the end of follow-up and 4 patients (10%) were alive with disease.

**Table 1:** The clinicopathologic features of the 40 Xp11.2 tRCC patients.

Case	Gender	Laterality	Tumor size (cm)	TFE3 IHC	TNM stage	AJCC stage	Nuclear grade	Fusion partner	Metastasis or recurrence status	Follow-up	Outcome
1	F	R	3.9	3+	T1aN1M0	3	4	ASPL	Bone metastasis after 48 months	62	DOD
2	M	R	4	2+	T1aN0M0	1	3	NONO	–	152	NED
3	M	L	3	3+	T1aN0M0	1	2	ASPL	–	172	NED
4	F	R	8.6	3+	T3cN1M0	3	3	ASPL	Liver metastasis after 2 months	33	DOD
5	F	R	13	3+	T3cN1M0	3	2	ASPL	Liver and brain metastasis after 12 months	25	DOD
6	M	R	6	2+	T1bN0M0	1	3	PRCC	Lung metastasis after 11 months	75	DOD
7	F	R	6	1+	T1bN0M0	1	3	ASPL	–	121	NED
8	F	R	5.8	3+	T3bN0M0	3	3	SFPQ	Lung metastasis after 7 months	15	DOD
9	M	L	3.7	3+	T1aN0M0	1	2	NONO	–	86	NED
10	F	R	7.1	3+	T2aN0M0	2	3	ASPL	–	86	NED
11	F	R	5	3+	T1bN0M0	1	2	PRCC	–	106	NED
12	F	R	3.5	2+	T1aN0M0	1	2	PRCC	Liver metastasis after 55 months	71	DOD
13	F	R	4.5	3+	T1bN0M0	1	3	PRCC	–	79	NED
14	F	L	12.4	3+	T3aN1M0	3	2	PRCC	Local recurrence after 12 months, peritoneal, LN, lung metastasis after 43 months	45	DOD
15	F	L	9.5	3+	T2aN0M0	2	2	PRCC	Local recurrence after 14 months, peritoneal metastasis after 32 months	39	DOD
16	M	L	3	2+	T1aN0M0	1	3	PRCC	–	71	NED
17	M	R	3	2+	T1aN0M0	1	2	NONO	–	54	NED
18	F	L	3.8	3+	T1aN0M0	1	2	NONO	–	47	NED
19	M	L	5.4	1+	T1bN0M0	1	2	SFPQ	–	45	NED
20	M	L	2.5	2+	T1aN0M0	1	3	NONO	–	39	NED
21	F	L	3	2+	T3aN0M0	3	3	NONO	–	40	NED
22	F	L	5	3+	T1bN0M0	1	1	SFPQ	–	38	NED
23	M	L	5.5	3+	T4N1M0	4	4	ASPL	Liver and LN metastasis at diagnosis	5	DOD
24	F	L	2.2	3+	T1aN0M0	1	2	SFPQ	–	28	NED
25	M	L	4	1+	T3aN1M0	3	4	PRCC	LN and vertebral column metastasis after 135 months	148	DOD
26	M	L	3.5	3+	T1aN0M0	1	3	NONO	–	18	NED
27	F	L	3	3+	T1aN0M0	1	3	PRCC	–	28	NED
28	M	L	2.6	2+	T1aN0M0	1	3	PRCC	–	27	NED
29	F	R	10	1+	T2aN0M0	2	2	ASPL	Vertebral column and soft tissue metastasis after 18 months	32	AWD
30	F	R	3.8	3+	T1bN1M0	3	3	ASPL	LN metastasis at diagnosis, local recurrence after 18 months	30	AWD
31	M	L	3.1	3+	T1aN0M0	1	3	SFPQ	–	20	NED
32	F	R	6	3+	T1bN0M0	1	2	SFPQ	–	16	NED
33	M	R	6.5	3+	T1bN0M0	1	3	SFPQ	–	31	NED
34	M	R	6.5	3+	T1bN1M0	3	3	SFPQ	LN metastasis at diagnosis, abdominal wall metastasis after 8 months	29	AWD
35	M	L	3	3+	T1aN0M0	1	3	ASPL	–	11	NED
36	F	L	16.5	3+	T4N1M0	4	4	ASPL	LN metastasis at diagnosis	13	AWD
37	M	R	4	2+	T1aN0M0	1	3	SFPQ	–	26	NED
38	F	R	4.4	3+	T1bN0M0	1	2	SFPQ	–	8	NED
39	F	L	6.5	3+	T1bN0M0	1	1	SFPQ	–	2	NED
40	F	R	5.5	2+	T3aN0M0	3	3	PRCC	–	1	NED

### 3.2 Pathology and molecular results

**Figure 1:**
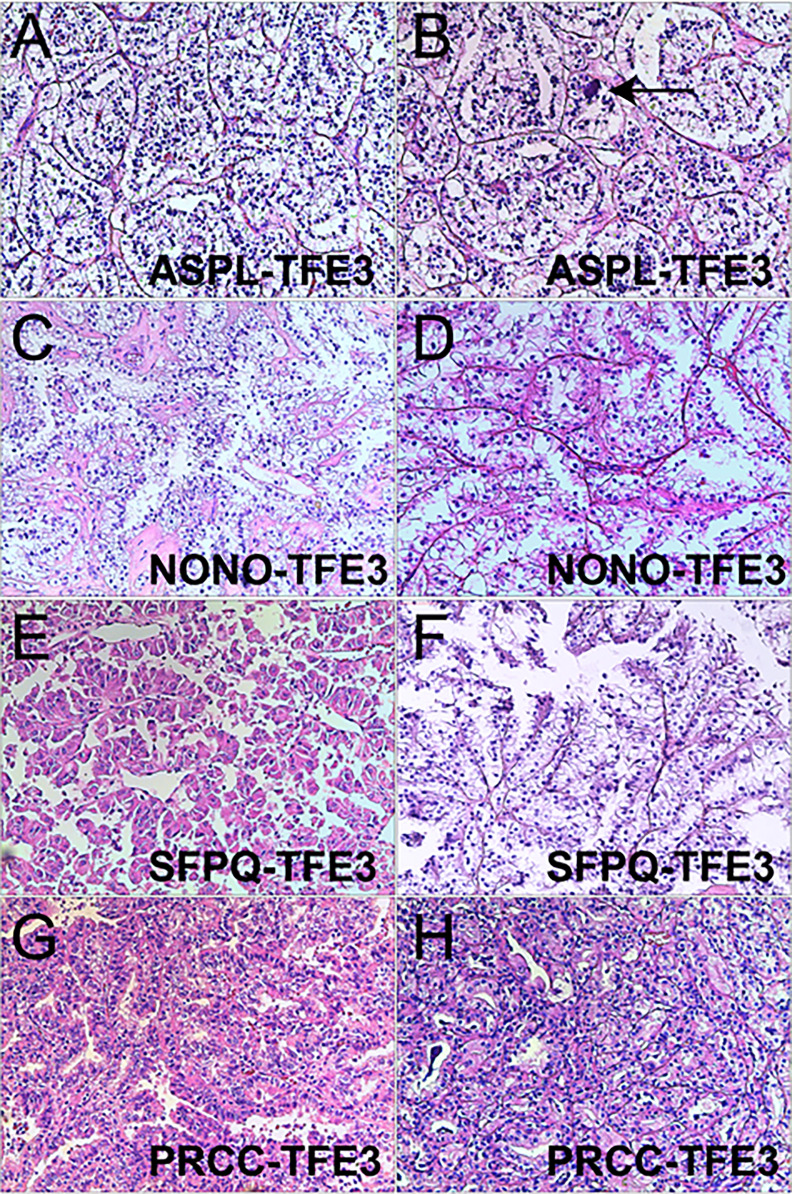
Representative images of morphologic features in Xp11.2 tRCCs with four common fusion subtypes. **(A, B)** ASPL-TFE3 cases showed nested architecture, clear to eosinophilic cells with voluminous cytoplasm and round nuclei, Psammoma bodies were frequently seen (arrowhead). **(C, D)** NONO-TFE3 cases showed papillary architecture with clear to flocculent eosinophilic cytoplasm. **(E, F)** SFPQ-TFE3 cases showed papillary architecture. **(G, H)** PRCC-TFE3 showed compact architecture with less voluminous cytoplasm. Original magnification: × 100 **(A–H)**. H&E: hematoxylin and eosin.

**Figure 2:**
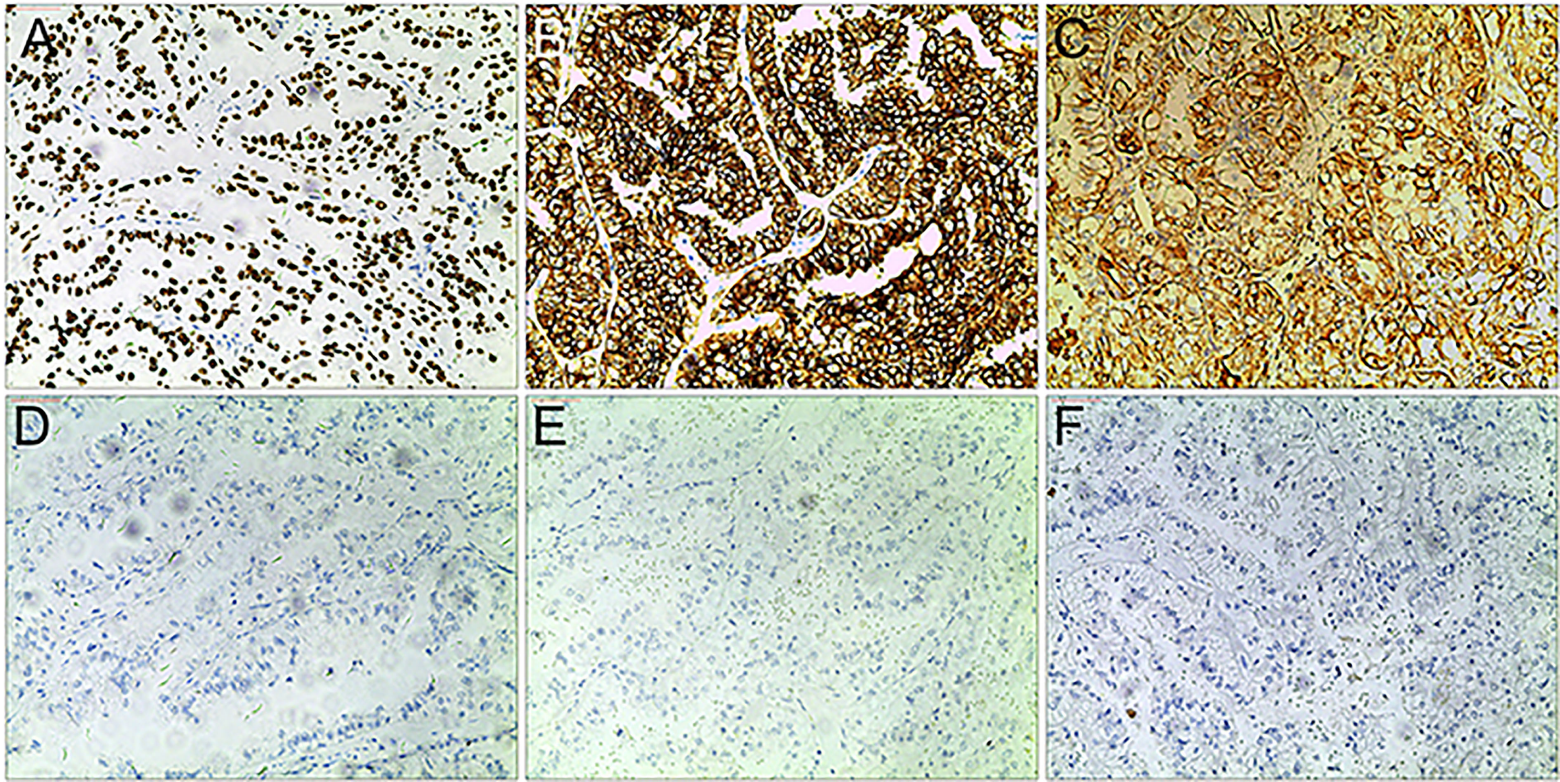
Representative IHC images of Xp11.2 tRCCs. **(A–C)** positive results of TFE3, CD10 and Vimentin. **(D–F)** Negative results of cathepsin K, CD117 and CA-IX. Original magnification: × 100 **(A–F)**.

**Figure 3:**
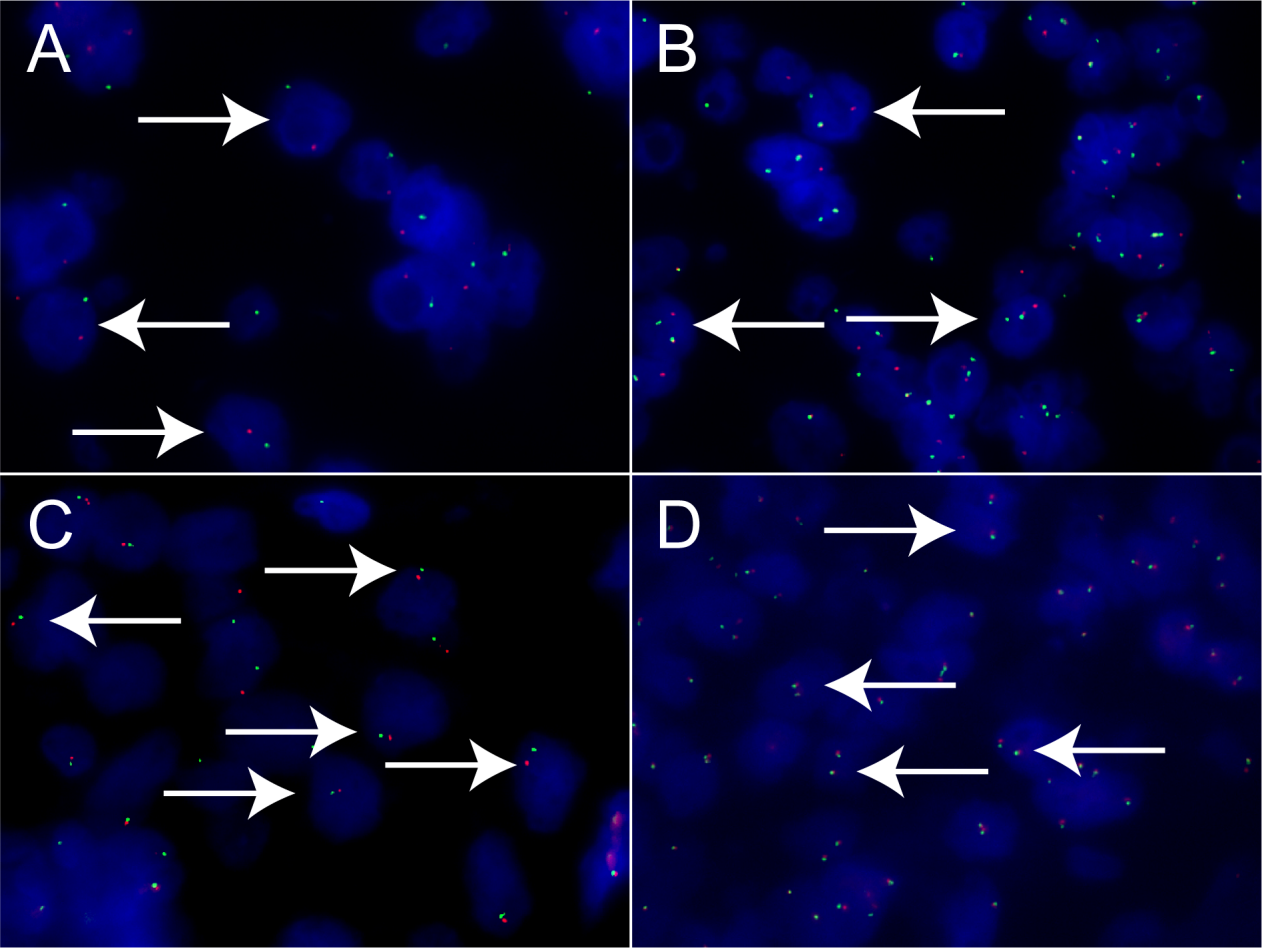
Results of TFE3 break-apart FISH in Xp11.2 tRCC. **(A, B)** Typical signal patterns in a male case (1R1G) and a female case (1R1G1F). **(C, D)** Equivocal signal patterns of NONO-TFE3 in a male case (1R1G) and a female case (1R1G1F). Original magnification: × 1000 **(A–D)**. R, red; G, green; F, fusion; FISH, fluorescence *in situ* hybridization.

Xp11.2 tRCC showed variable morphological characteristics according to different fusion types. The typical feature of Xp11.2 tRCC was the presence of papillary, glandular, nested, or tubular architectures with clear or eosinophilic cytoplasm, psammoma bodies or calcification were occasionally seen. Papillary architecture was the most common morphology in the 40 cases. Nested architecture and pseudorosettes were seen in 4 SFPQ-TFE3 cases and 5 ASPL-TFE3 cases while none of cases with NONO-TFE3 and PRCC-TFE3 showed this architecture. The most distinctive feature of PRCC-TFE3 cases was the compact (Closely arranged tumor cells with less voluminous cytoplasm and few psammoma bodies) architecture (7/11,63.6%), which was quite different from the other subtypes. Psammoma bodies appeared the most in ASPL-TFE3 cases (8/11, 72.7%) while they were rarely observed in NONO-TFE3 (1/7, 14.3%) and PRCC-TFE3 cases (0/11, 0%). The histologic features of Xp11.2 tRCC with four main fusion types were shown in **Figure 1**. In term of IHC profiles, all the cases showed TFE3 nuclear positivity and the majority of Xp11.2 tRCC cases (90%) showed moderate (++) to strong (+++) positivity. Cathepsin K were seen predominantly in PRCC-TFE3 cases (7/11.63.6%), which is useful to distinguish PRCC-TFE3 cases from the other types. CD10 was positive in most of the cases (34/40, 85%) and half of the cases (22/40, 55%) showed vimentin positivity. The positivity of CA-IX, CD117 and CK7 is uncommon. The morphological and IHC profiles of 40 cases were shown in **Table 2**. In FISH results, 33 cases showed typical translocation signals and 7 cases with NONO-TFE3 fusion showed equivocal signals (split but adjacent signals). The representative IHC and FISH features were shown in **Figures 2**, **3**. Gene fusions were verified by RNA sequencing, of which the gene fusions involve ASPL-TFE3 gene fusion (11 cases), PRCC-TFE3 gene fusion (11 cases), SFPQ-TFE3 gene fusion (11 case) and NONO-TFE3 gene fusion (7 cases). The representative sequencing results were shown in **Table S1**.

**Table 2:** Morphological and IHC features of the 40 Xp11.2 tRCC patients.

Item	SFPQ(n=11)	NONO(n=7)	ASPL(n=11)	PRCC(n=11)
**Morphological features, n (%)**				
Papillary architecture	6 (54.5)	5 (71.4)	3 (27.3)	3 (27.3)
Solid/nested architecture	4 (36.3)	0 (0)	5 (45.4)	0 (0)
Compact architecture	1 (9.1)	0 (0)	0 (0)	7 (63.6)
Variable morphologies	0 (0)	2 (28.6)	3 (27.3)	1 (9.1)
Psammoma bodies	5 (45.4)	1 (14.3)	8 (72.7)	0 (0)
**IHC-positive results, n (%)**				
TFE3	11 (100)	7 (100)	11 (100)	11 (100)
Cathepsin K	0 (0)	0 (0)	1 (9.1)	7 (63.6)
CD10	10 (90.9)	6 (63.6)	9 (81.8)	9 (81.8)
CA-IX	2 (18.2)	0 (0)	1 (9.1)	1 (9.1)
Vimentin	6 (54.5)	3 (27.3)	6 (54.5)	7 (63.6)
CD117	2 (18.2)	0 (0)	1 (9.1)	0 (0)
CK7	1 (9.1)	0 (0)	0 (0)	0 (0)

### 3.3 The differences between DBHS family group and non-DBHS family group

Among all the 40 cases, 11 cases with SFPQ-TFE3 gene fusion and 7 cases with NONO-TFE3 gene fusion were classified in DBHS group, the remaining cases with ASPL-TFE3 or PRCC-TFE3 gene fusion were classified in non-DBHS group (**Table 3**). No statistical difference was seen in the baseline clinicopathologic characteristics, including age, gender, laterality, tumor size, pT stage and AJCC stage (P >0.05) between DBHS family group and non-DBHS family group. However, there was a significant difference in pN stage (P = 0.027) at surgery and M stage at the last follow‐up (P = 0.009). Furthermore, although no statistical difference was found in nuclear grade and follow-up time (all P >0.05), cases in non-DBHS family group tend to have a worse outcome (P =0.013).

**Table 3:** Comparison of clinicopathological features between DBHS family group and non-DBHS family group.

Items	Total (n=40)	DBHS family group (n=18)	Non-DBHS family group (n=22)	P value
**Age (years)**				0.915
Median (range)	35.5 (7-70)	34.5 (24-55)	35.5 (7-70)	
Mean ± SD	37.3 ± 13.4	37.6 ± 11.2	37.1 ± 15.2	
**Gender, (n, %)**				0.131
Male	17 (42.5)	10 (55.6)	7 (31.8)	
Female	23 (57.5)	8 (44.4)	15 (68.2)	
**Laterality, (n, %)**				0.525
Left	20 (50)	10 (55.6)	10 (45.5)	
Right	20 (50)	8 (44.4)	12 (54.5)	
**Tumor size (cm)**				0.177
Median (range)	4.5 (2.2-16.5)	4.0 (2.2-6.5)	5.25 (2.6-16.5)	
Mean ± SD	5.5 ± 3.1	4.38 ±1.44	6.3 ± 3.8	
**pT stage, (n, %)**				0.149
T1-T2	31 (77.5)	16 (88.9)	15 (68.2)	
T3-T4	9 (22.5)	2 (11.1)	7 (31.8)	
**pN stage (n, %)**				**0.027**
N0	31 (77.5)	17 (94.4)	14 (63.6)	
N1	9 (22.5)	1 (5.6)	8 (36.4)	
**M stage at the last follow‐up (n, %)**				**0.009**
M0	27 (67.5)	16 (88.9)	11 (50.0)	
M1	13 (32.5)	2 (11.1)	11 (50.0)	
**AJCC stage (n, %)**				0.096
I-II	28 (70.0)	15 (83.3)	13 (59.1)	
III-IV	12 (30.0)	3 (16.7)	9 (40.9)	
**WHO/ISUP grade, (n, %)**				0.243
Grade 1-2	16 (40.0)	9 (50.0)	7 (31.8)	
Grade 3-4	24 (60.0)	9 (50.0)	15 (68.2)	
**TFE3 IHC (n, %)**				0.683
+	4 (10.0)	1 (5.6)	3 (13.6)	
++	10 (25.0)	5 (27.8)	5 (22.7)	
+++	26 (65.0)	12 (66.7)	14 (63.6)	
**Follow-up (months)**				0.201
Median (range)	35.5 (1-172)	30 (2-152)	53.5 (1-172)	
Mean ± SD	49.4 ± 42.0	38.6 ± 34.3	58.2 ± 46.3	
**Outcome (n, %)**				**0.013**
Alive	30 (75.0)	17 (94.4)	13 (59.1)	
Dead	10 (25.0)	1 (5.6)	9 (40.9)	

Bold indicates P values less than 0.05.

### 3.4 Prognosis analysis for survival

**Figure 4:**
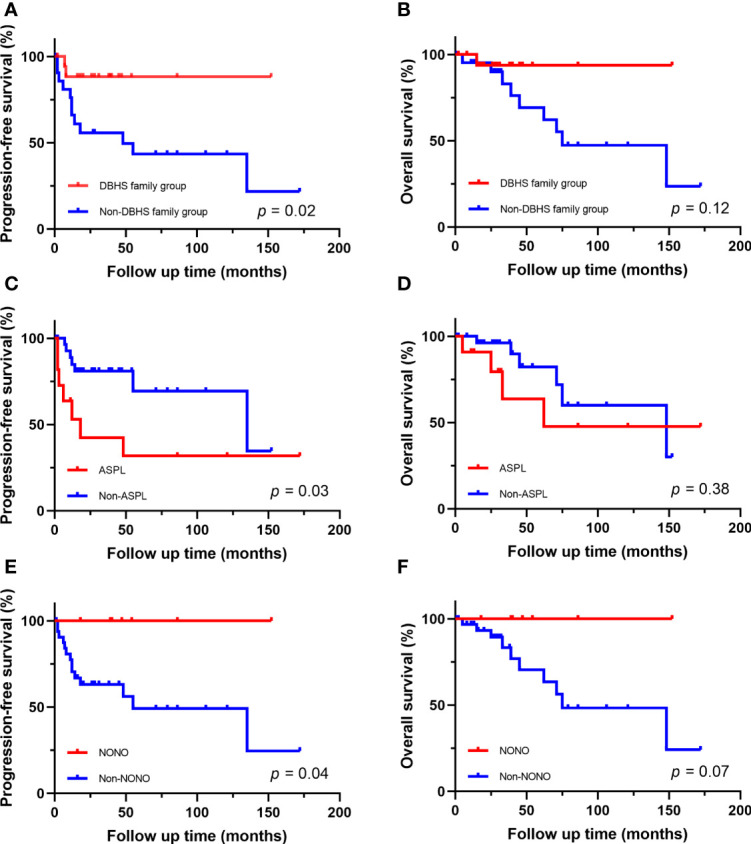
Survival analysis of different fusion partners. **(A, B)** PFS and OS for patients in DBHS famliy group and non-DBHS famliy group. **(C, D)** PFS and OS for patients with ASPL-TFE3 fusion and non-ASPL fusion. **(E, F)** PFS and OS for patients with NONO-TFE3 fusion and non-NONO fusion.

**Figure 5:**
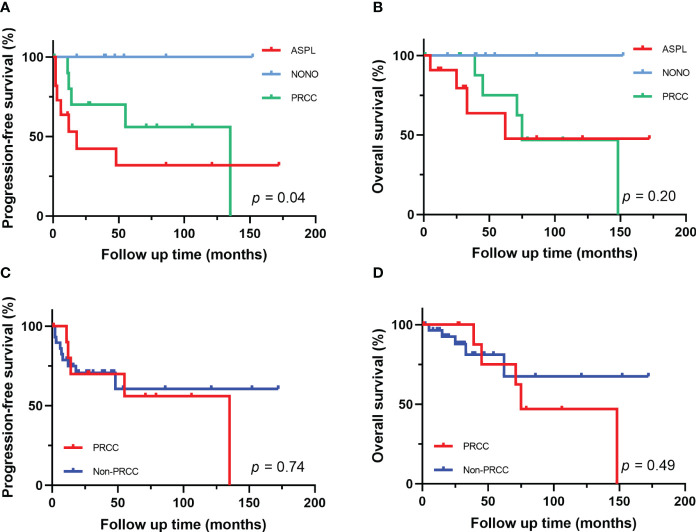
Survival analysis of different fusion partners. **(A, B)** PFS and OS for patients with different fusion subtypes. **(C, D)** PFS and OS for patients with PRCC-TFE3 fusion and non-PRCC fusion.

The follow-up time of the 40 Xp11.2 tRCC cases with the four main fusion types was compared. The median follow-up time was 35.5 months (range, 1–172 months). Survival analysis showed that cases in DBHS family group have better progressive-free survival (PFS) (median PFS: not reached vs. 48 months, P = 0.023, **Figure 4A**) and overall survival (OS) (median OS: not reached vs. 75 months, P = 0.115, **Figure 4B**) compared with those in non-DBHS family group. In the subgroup analysis for the four different fusion types, patients with ASPL-TFE3 fusion were associated with poor PFS compared with other subtypes (median PFS: 18 months vs. 135 months, P = 0.026, **Figure 4C**) even though there was no significant difference in OS (median OS: 62 months vs. 148 months, P = 0.379, **Figure 4D**). In contrast, patients with NONO-TFE3 fusion showed better PFS (median PFS: not reached vs. 55 months, P = 0.040, **Figure 4E**) while there remained no significant difference in OS (median OS: not reached vs. 75 months, P = 0.077, **Figure 4F**). The difference in follow-up between TFE3-SFPQ group and the other subtypes were not compared due to the short follow-up period of TFE3-SFPQ group. Survival analysis indicated that cases with ASPL-TFE3 fusion have the worst PFS among the other three subtypes with sufficiently long follow-up (median PFS: 18 months vs. not reached vs. 135 months, P = 0.04, **Figure 5A**) although there was no difference in OS (P >0.05, **Figure 5B**). No statistically significant difference was seen in PFS and OS between PRCC and non-PRCC group (P >0.05, **Figures 5C–D**).

## 4 Discussion

In recent years, with an in-depth understanding of the genomic spectrum of RCCs, Xp11.2 tRCC has received increasing attention. Apart from morphological and molecular features different from conventional RCCs, Xp11.2 tRCC itself is a group of highly heterogeneous tumors and various fusion partners has proven to be the source of this heterogeneity (12, 14, 23, 24). ASPL, PRCC and SFPQ were regarded as relatively common fusion partners initially while the pericentric inversion of NONO and TFE3 is also not rare nowadays. Therefore, in this study, we retrospectively investigated the clinicopathological features of 40 Xp11.2 tRCC cases with the above four fusion types in our cohort by IHC, FISH and RNA sequencing. The initial diagnosis of Xp11.2 tRCC was based on IHC and FISH analysis and then the fusion types were verified by RNA sequencing.

Xp11.2 tRCCs with different fusion subtypes have respective histological characteristics. As regards morphology, cases with ASPL-TFE3 fusion could present with various features including alveolar, papillary or nested architecture, which is not much different from other subtypes, but psammoma bodies were most common seen in ASPL-TFE3 cases among the four subtypes (25, 26). Papillary architecture frequently occurred in SFPQ-TFE3 subtype while pseudorosette-like architecture was occasionally described (27). NONO-TFE3 cases could also show a papillary architecture but it was more like the appearance of secretory endometrioid(7). PRCC-TFE3 cases tended to show a compact architecture and psammoma bodies were rare (28). In addition, recent literature reported the distinctive morphology of MED15-TFE3 cases. This subtype often showed a multicystic architecture without solid composition, resembling the feature of multilocular cystic renal cell neoplasm of low malignant potential (MCRN-LMP) (29). In regard to IHC, moderate (++) to strong (+++) TFE3 nuclear positivity is the primary clue to the initial diagnosis of Xp11.2 tRCC and cathepsin K positivity seems to be relevant to PRCC-TFE3 subtype. CA-IX and CK7 were always negative in Xp11.2 tRCC, which could help to exclude clear cell RCC (ccRCC) and papillary RCC (PRCC) (30). TFE3 break-apart FISH was the most effective method to detect TFE3 rearrangement in clinical practice (31), while equivocal or false-negative split signal pattern could be observed in several special fusion subtypes such as NONO(19) and RBM10(6). Hence, suspicious Xp11.2 tRCC cases with negative FISH results should be confirmed by further sequencing.

The fusion gene partners are likely to have a functional role in the oncogenesis of Xp11.2 tRCC and the underlying mechanisms may influence clinical behavior(2). SFPQ and NONO, belonging to DBHS family, are both pre-mRNA splicing factors and associated with tumorigenesis of multiple cancers such as prostate cancer and breast cancer (16–18, 32). Due to the homology of SFPQ and NONO gene, we classified Xp11.2 tRCC harboring SFPQ-TFE3 and NONO-TFE3 as a group and compared the characteristics of this group with non-DBHS family group (cases with PRCC-TFE3 and ASPL-TFE3 fusion). In the DBHS family group, only one patient (5.6%) was found lymph node (LN) metastasis in surgery, while 8 patients (36.4%) in non-DBHS family group had LN metastasis. This phenomenon suggested that cases in DBHS family group tend to show more lymph node metastasis in the early stages of disease. More importantly, half of the cases (50%) in non-DBHS family group showed local recurrence or distant metastasis at the last follow‐up, which demonstrated that cases in non-DBHS family group were likely to display a more aggressive and invasive behavior compared with cases in DBHS family group. It is worth mentioning that previous literature reported that Xp11.2 tRCC with ASPL-TFE3 fusion are more prone to present at advanced stage than cases with PRCC-TFE3 (14), however, in our cohort, although positive lymph node status at surgery is more common in ASPL-TFE3 cases (6/11, 54.5%), there was no statistically significant difference in pM stage at the last follow‐up between ASPL-TFE3 and PRCC-TFE3 cases. In addition, cases 25 presented with LN and vertebral metastasis after 10 years, which indicated follow-up period need to be long enough to estimate metastasis status and outcome.

The prognosis of Xp11.2 tRCC could be affected by a variety of factors. Above all, the impact of age on prognosis is prominent in Xp11.2 tRCC. Pediatric patients tended to show an indolent course whether the presence of lymph node metastasis (33). Case 3 in our cohort is a 7-year-old boy and showed no evidence of disease after 172-month follow-up, which seemed to support this view. Recent study has demonstrated that pediatric patients with Xp11.2 tRCC have a lower burden of genetic alteration compared with adult patients (34) and this could be a probable explanation of this phenomenon. On the contrary, Xp11.2 tRCC was more aggressive in adults, older age and distant metastasis were two predictors of poor prognosis (14, 35). Beyond that, the correlation between fusion subtypes and outcomes are currently being explored. In our study, cases with ASPL-TFE3 fusion showed a worse PFS compared with non-ASPL group, which supported that ASPL-TFE3 fusion may represent a more adverse prognosis in previous studies (12, 14, 36). Apart from fusion subtypes, copy number alterations (CNA) and chromosomal amplification could also affect the prognosis of Xp11.2 tRCC (23, 24). Patients with CNA burden had worse survival outcomes and 22q loss was an independent adverse prognostic marker (12, 37).In addition, a recent proteogenomic study revealed that deletions of 3p could lead to decreased OS *via* trans- effect or cis-effect (36). Overall, genetic alteration is an important cause of the clinical heterogeneity in Xp11.2 tRCC.

The effective treatment strategy for Xp11.2 tRCC is still unclear. Several clinical investigations have indicated that Xp11.2 tRCC patients had a poor response to immune checkpoint inhibitors (ICIs) and tyrosine kinase inhibitors (TKIs) (38, 39). However, the response to immunotherapy and vascular endothelial growth factor receptor (VEGFR)-targeted therapy seemed to vary according to the fusion subtypes. A previous case report demonstrated an 18-year-old male Xp11.2 tRCC patient with ASPL-TFE3 fusion had a favorable response to sorafenib (40). Recent transcriptomic analysis also revealed tumors with ASPL-TFE3 fusion are more likely to benefit from antiangiogenic treatments compared with the other subtypes and ICI plus TKI combination therapy may be a better choice (12). Besides, due to the activation of RPS6KB1 in Xp11.2 tRCC, Trilaciclib, an FDA-approved CDK5 inhibitor, was expected to become a potential therapeutic drug (36).

There are some limitations to our research. Firstly, although we have collected as much data of Xp11.2 tRCC cases as possible to fulfill the research goal, the sample size still needs to be further expanded. Secondly, our follow-up data only suggested there was a significant difference in PFS, therefore the patients require a longer follow-up time to validate the results.

In summary, Xp11.2 tRCC is a group of highly heterogeneous tumors. Of the four common fusion subtypes, cases in non-DBHS family group more frequently developed LN and distant metastases than cases in DBHS family group. Tumors with ASPL-TFE3 fusion tend to have a worse outcome while those with NONO-TFE3 fusion exhibit a relatively good prognosis. Novel therapeutic approaches and targets for Xp11.2 tRCC with different fusion subtypes remain to be explored.

## Data availability statement

The original contributions presented in the study are included in the article and **Supplementary Material**. The Illumina HiSeq next generation sequencing was performed by GloriousMed. The datasets presented in this article are not readily available because patient confidentiality and participant privacy, thus requests to access the datasets should be directed to info@gloriousmed.com.

## Ethics statement

The studies involving human participants were reviewed and approved by Institutional Review Board of the Nanjing Drum Tower Hospital. The patients/participants provided their written informed consent to participate in this study.

## Author contributions

WGu and YZ performed study concept and design; YZ and XP performed developpment of methodology and writing, review and revision of the paper; WGu provided acquisition, analysis and interpretation of data, and statistical analysis; WGa and HG provided technical and material support. All authors read and approved the final paper.
